# Prediction of Ligand Binding Using an Approach Designed to Accommodate Diversity in Protein-Ligand Interactions

**DOI:** 10.1371/journal.pone.0023215

**Published:** 2011-08-10

**Authors:** Lorraine Marsh

**Affiliations:** Department of Biology, Long Island University, Brooklyn, New York, United States of America; University of Akron, United States of America

## Abstract

Computational determination of protein-ligand interaction potential is important for many biological applications including virtual screening for therapeutic drugs. The novel internal consensus scoring strategy is an empirical approach with an extended set of 9 binding terms combined with a neural network capable of analysis of diverse complexes. Like conventional consensus methods, internal consensus is capable of maintaining multiple distinct representations of protein-ligand interactions. In a typical use the method was trained using ligand classification data (binding/no binding) for a single receptor. The internal consensus analyses successfully distinguished protein-ligand complexes from decoys (*r* ^2^, 0.895 for a series of typical proteins). Results are superior to other tested empirical methods. In virtual screening experiments, internal consensus analyses provide consistent enrichment as determined by ROC-AUC and pROC metrics.

## Introduction

The scoring or classification of small molecule binding to a receptor or enzyme is an important problem for many areas of biology including drug development for therapeutics However it has proven difficult to accurately predict ligand interactions by any single method [1], [2]. Two types of methods are commonly used. Docking functions are simplified (and less accurate) methods used in the process of docking molecules. Speed is a primary concern for docking functions such as the Vina docking function [3]. Scoring functions are intended to be more accurate and used to study smaller groups of potential complexes [4]–[6].

Physics-based scoring function approaches record features of ligand-receptor interactions and sum well-established energetic terms such as Van der Waals interactions, charge interactions, hydrogen bonding etc. The AMBER and CHARMm force fields and the MM-PBSA and MM-GBSA methods are examples of this approach [7]. By adding a quantum treatment to the analysis of interactions (as in free energy perturbation, FEP [8]), it is possible in principal to very accurately predict ligand affinities, but in practice, such methods are slow, and still are subject to computational uncertainties. An advantage of physics-based approaches is that the equation describing binding in one complex should be the same as the equation describing any other complex. Binding of a specific protein-ligand pair is analyzed in the context of broad-based rules.

Another group of approaches for determining protein-ligand affinity is the *knowledge-based* group of methods. These extract the probability of specific atomic interactions occurring in observed (x-ray crystallographic) complexes and treat these probabilities, following a Boltzmann approach, as reflecting energy of interaction [5]. Drugscore is a knowledge based function [5]. Again the basis of binding determination is very broad, reflecting knowledge derived from atom interactions in many environments.

Empirical potential scoring functions follow the physics-based model but add additional, terms for molecular interactions and parameterize the resulting affinity equation. Terms are adjusted by regression of a linear equation describing interactions to train the method to produce observed ligand affinities as in X-score [6]. Alternatively the equations can be optimized in other ways as in Vina score [3]. Empirical methods are typically trained on a set of protein-receptor complexes or on ligand complexes with a specific protein. As such, empirical methods are more focused on specific protein-receptor interactions than physics-based or knowledge-based methods. Most empirical methods derive from the early method ChemScore [3]. They have a small number of factors and are trained by linear regression as described.The internal consensus analysis approach presented here is an empirical potential method with conceptual similarities to Vina and X-score, but with novel features including an extended set of factors and analysis by neural network that duplicate the functionality of consensus methods.

 One factor that makes scoring ligand affinity difficult is that various ligand binding sites may present different types of potential interactions. Also, various ligands may bind a given protein in different modes, using different portions of the binding site. One way to adapt to the variety of different types of ligand binding is to form a consensus amongst methods that might have strengths with one type of complex or another. Consensus methods for scoring protein-ligand binding have found widespread use. An example is the averaging of three hydrophobic terms in X-score [6]. Another use of the consensus is to improve representation of the diversity present in complex data [9], [10]. The advantage of consensus schemes is that the specific weaknesses of individual methods may be overcome. The disadvantage is that an analysis especially suited for a class of ligand or receptor may lose that advantage when its output is mixed with that of other methods. Also, computation becomes more complicated and less interpretable. Ideally, a method might allow the power associated with consensus methods in a easily trainable and flexible form.

Neural networks are an attractive option for creating consensus [11], [12]. Neural networks in particular have the ability to learn mixtures of distinct patterns [13]. This learning should permit neural network identification of protein-ligand complexes of different types, such as complexes dominated by hydrogen bonds and complexes dominated by hydrophobic interactions. Almost all existing methods merge these very different patterns into a single type for scoring [3], [6], [14]. Ideal physics-based methods can, in principle, correctly analyze disparate types of complexes without the need for neural network-type analysis [8]. However these methods currently are limited by speed considerations.

Virtual screening is the identification of novel ligands that might bind a binding site, using only computation [15], [16]. Virtual screening represents a challenge for computational methods because of the impreciseness of current scoring functions. There are two main types of virtual screening, ligand-based and receptor-based. Ligand-based methods are based on finding new ligands similar in key respects to existing ligands. Receptor-based methods are based on finding molecules that are capable of binding to a receptor binding site. Receptor-based methods have shown the potential to find completely novel ligands [17]–[19]. The success of receptor-based methods is dependent on the ability to accurately classify virtual ligands based on whether or not they have the potential to bind tightly to a binding site. The true affinity of the computationally selected ligands can then be determined by laboratory analysis.

Here we present a method for predicting the relative affinity of ligands bound to protein binding sites. The method is conceptually an empirical potential approach but is nonlinear, with more input factors than the typical empirical method. The extra terms are included to mimic the larger number of factors that are typically observed in consensus methods. The inclusion of a neural network also allows the analysis to robustly work with groups of protein-ligand complexes of diverse characteristics. This feature, robustness with diverse types of binding site, is also typical of consensus methods. Internal consensus analysis works well on many proteins and in a variety of types of protein-ligand interaction studies. Its features could easily be incorporated into other scoring applications.

## Results and Discussion

### Overview of the internal consensus method

The method has two or three basic steps plus some elaborations. Step 1) involves assaying a protein-ligand complex using 9 factors that include features such as contacts and hydrogen bonds. The structure of the complex and additional information about atom types and charge are used to determine these 9 values. Step 2) involves using the 9 factors to input to a neural network, which in turn outputs a score value. The score values can be used for analysis, e.g. to calculate an AUC value, or directly as a prediction of whether a complex is stable or not. Step 3) is training and is only needed for new types of complexes. Step 3) uses data from Step 1) for a curated set of complexes to determine coefficients that optimize the function of the neural network.

### Factors for use in forming an internal consensus to predict protein-ligand binding

Much scoring of protein-ligand binding complexes by consensus methods has been ad hoc and based on combining the output of existing applications [20]. As a matter of observation, the approach has led to improved scoring [20] but the mechanism of improvement has been unclear. The most obvious possibility is that the increased number of factors and parameters associated with combining disparate methods leads to a more complex model for ligand binding and hence an improvement in data fitting. It seemed that a similar result could be achieved by deliberately starting with a more complex model [6], [9] or by factoring in the diversity of the data [10]. The present model includes both approaches. First, more than one factor was scored for each of the major contributions to energy of ligand binding. These factors were chosen to be different, but to correspond with major lines of thinking about binding interactions [1].

For three categories of interaction, different factors for inclusion in scoring protein-ligand interactions were selected. For contact interactions, Van der Waals energies, one factor was constructed to resemble a classic Lennard-Jones function. The other was designed to reward more distant interactions with a conformational ensemble model of protein-ligand complexes in mind (for example [21]). For hydrogen bonds, one factor defined hydrogen bond energy solely on distance, whereas the other considered bond angle as well. A negatively weighted factor was based on potential hydrogen bond donors or acceptors that lacked an apparent partner (‘frustrated’ hydrogen bonds). A fourth factor included potential distant hydrogen bonds. These could be formed via an intervening water molecule, or reflect imprecision of the protein-ligand complex coordinates, considering again a conformational ensemble. For hydrophobic interactions, one factor considered interactions of the ligand with hydrophobic amino acid residues of the receptor, while the other factor considered interactions on the atomic level. Like some of the other factors these two factors allow weighting for accurate models or less precise docking. For coulombic charge interactions only a single factor was used. We wanted to include the possibility that the dielectric constant in binding sites could vary, but recognized that simple weighting of the single factor could achieve this goal.

**Figure 1:**
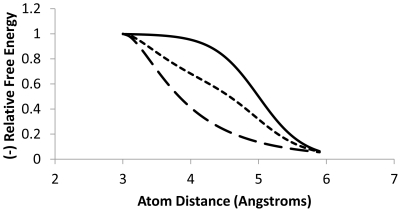
Combinations of factors. Combining factors in varying proportions can effectively produce novel factors during training that are functions of the original factors. VDW1, VDW2 and a hybrid factor are shown as a function of atom distances. Dashed line, distance function of factor VDW1; solid line, function of factor VDW2 and dotted line, a 1∶1 mixture (coefficients of VDW1 and VDW2 both set to fraction 0.5). Free energy values are scaled to the range 0–1. Energy values are presented for an atom pair with each atom assuming a VDW radius of 1.5 Angstroms.

These factors allow a very flexible scoring of protein-ligand interactions, akin to that achieved with consensus methods that rigidly combine different scoring applications. Figure 1 shows a pair of factors based on Van der Waals (VDW) interactions and the way that weighted combinations can create a custom family of scoring functions. Of note, the 9 parameters used for the internal consensus analysis presented here encompass only the known physical factors of ligand binding. The VDW, hydrogen bond, and hydrophobic terms are very similar to those of other scoring functions including X-score etc. [6]. Some scoring methods neglect charge interactions [3], however for some proteins, such as trypsin they contribute significantly to ligand binding scoring (see below).

### Correlation of factors and effective number of parameters

Since some of our factors were chosen deliberately to reflect similar underlying aspects of energetics of protein-ligand interactions, it was of interest to determine correlation amongst the factors. The 9 factor values for the 39 proteins of the DUD database were analyzed. Overall there is significant correlation for many of the factors (Table 1). Some of the conceptually related factors are correlated (VDW factors, H-bond factors with and without angles), but, interestingly, not all.

**Table 1:** Factor-factor scoring correlation for a mixture of proteins.

	**Vdw**	**Vdw2**	**HB**	**HBang**	**HBlon**	**NHB**	**Hydr1**	**Hydr2**	**Coul**
Vdw	1.00	0.75	−0.76	−0.54	−0.39	−0.04	0.26	0.54	0.07
Vdw2	0.75	1.00	−0.28	−0.25	−0.05	−0.05	0.29	0.67	0.10
HB	−0.76	−0.28	1.00	0.81	0.61	0.10	−0.06	−0.11	−0.01
HBang	−0.54	−0.25	0.81	1.00	0.60	0.22	0.14	0.08	−0.06
HBlong	−0.39	−0.05	0.61	0.60	1.00	0.35	0.25	0.21	−0.08
NHB	−0.04	−0.05	0.10	0.22	0.35	1.00	0.44	0.21	−0.18
Hydr1	0.26	0.29	−0.06	0.14	0.25	0.44	1.00	0.51	−0.10
Hydr2	0.54	0.67	−0.11	0.08	0.21	0.21	0.51	1.00	−0.03
Coul	0.07	0.10	−0.01	−0.06	−0.08	−0.18	−0.10	−0.03	1.00

The 9 factors (and the neural network architecture described below) lead to a potentially high number (50) of parameters for the internal consensus model. This, in turn, could weaken analyses or lead to overfitting. Typical consensus methods also have a high number of parameters, though distributed in the component methods used to form the consensus. Correlation of the factors used to form an internal consensus could simplify the internal consensus model. The number of effective parameters in practice [22] was calculated using the expression:
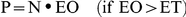
Where P is the effective number of parameters; N is the number of training sets; EO is the observed error in scoring; and ET is the error rate achieved during training. The effective number of parameters on various data sets was 14.7+/−2.5. Thus the internal consensus model in practice has significantly fewer than the maximum number of parameters, reducing, somewhat, the amount of data required to make predictions. For this study, the amount of training data was sufficient to permit 90%–99% accuracy of the method. Accuracy is defined here based on ROC AUC measures of complex prediction accuracy described further below.

### Neural network for representation of multiple models for ligand binding

Neural networks have special advantages in classification of complex data. In particular they can learn to recognize correct targets embedded in data with various types of patterns. Much effort has been spent in the attempt to create a universal scoring method applicable to all proteins. Others have suggested that superior pragmatic results can be achieved using machine-learning methods that recognize the special features of a group of proteins [10]. Neural networks are well-adapted to the latter approach. The neural network used in the internal consensus analysis has 9 input nodes each taking one factor (plus one bias input node), 5 hidden nodes (plus one bias hidden node) and 1 output node corresponding to the prediction. The network has full connectivity between input and hidden layer nodes and hidden layer and output nodes. It is trained using backpropagation [13]. The 5 hidden nodes can each specialize in a type of protein-ligand interaction found in the training set. In practice 3–5 hidden nodes have significant weight after training with the data sets of this work suggesting that no single regression might fully capture the binding patterns present.

Good ligands all show high affinity for their binding sites, but the mechanisms for achieving that high affinity vary. Neural networks and consensus methods reflect two different approaches to permit machine learning to represent multiple patterns for ligand binding. Those specific patterns might include ligands that achieve high affinity mostly through hydrogen bonding and charge interactions and ligands that achieve high affinity mostly through hydrophobic interactions. Neural network architecture, used in internal consensus, is ideal for holding these multiple representations in a single model. Consensus models achieve similar results indirectly by combining methods that are strong in analysis of one type of complex or another.

Many methods exist to produce a numerical score for ligand-receptor complexes. Often this score is interpreted as related in some way to ΔG of ligand binding [3], [6], [14]. The internal consensus neural network instead produces a nonlinear binding score not directly comparable to free energy. Also, in this work, the analysis has been trained using discrete data in which binding was scored as non-binding vs. binding, rather than using continuous ligand affinity data. There are several good reasons to take this approach. Some binding data is corrupted and unreliable because it was assembled from several sources [23]. Generally binding affinities from a single source with a single method are highly reliable in ranking the affinity of ligands for a protein target [23]. But heterogeneous data is less reliable and leads to training errors for methods dependent on such data. On the other hand the internal consensus method is trained based on comparison of known binding molecules (often approved drugs or established probes with affinities between 10 nM and 10 mM) to decoys which have a low probability of being binders. Thus the discrete type of data used to train here might even be more reliable in some ways than the continuous data more commonly used. Only more experience will answer that point, but discrete data is more widely available, permitting more focused training.

**Figure 2:**
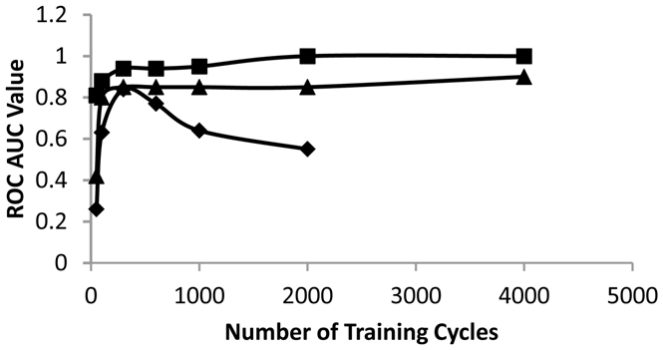
Neural network training speed. The accuracy of internal consensus predictions is compared to the number of training cycles. Overtraining is evident in the curve in which accuracy drops after an increase in training cycles. Squares, trypsin; triangles, HIV-1 protease; diamonds, DUD database set of proteins.

Neural network training consists of finding a local error rate minimum after iterative training cycles [13]. One area of concern is overfitting, in which the network overlearns irrelevant details at the expense of generalizable patterns. To test for overfitting the neural network was trained for varying numbers of cycles then tested with examples not part of the original training set. Figure 2 shows that the training speed depends on the particular data being studied. The trypsin and HIV protease data trained slightly more quickly than the mixed native data set. The mixed native complex set trained well at 300 cycles and overtrained at higher numbers of cycles. For the other proteins, the optimal number of cycles was reached at 300 or fewer cycles, but overtraining was not prominent. For this work, an optimal number of training cycles to facilitate accurate determination but avoid overfitting was determined for each type of experiment. Overfitting was most often a problem when training on a mixture of proteins with different features. Overfitting in all cases could be minimized by reducing the number of training cycles. It is important to note that, with the experimental arrangement used here, overfitting would only act to worsen, never to improve the accuracy of the method. This is because the training dataset and scoring dataset had no members in common so overfitting would lead to mistraining.

### Ability of internal consensus to distinguish native ligands from decoys

An internal consensus protocol was performed on a series of native ligand/receptor complexes. In each case native ligands were matched to 5 DUD decoys that have similar sizes and characteristics to the high affinity ligands but are predicted to bind with much lower affinity [24], [25]. Decoys were docked using Vina [3]. Three groups were analyzed: trypsin (73 ligands), HIV protease (112 ligands) [5] and DUD natives (39 proteins with 1 ligand/protein). Vina was used as a comparison method. Vina has been shown to successfully predict free energy of binding of ligands [4]. Vina is related to X-score [6], but has been trained on a larger sample of receptor-ligand complexes and uses more scoring factors. Vina uses a model that employs a single set of parameters for all complexes, whereas the other analyses here are trained on specific datasets.

**Figure 3:**
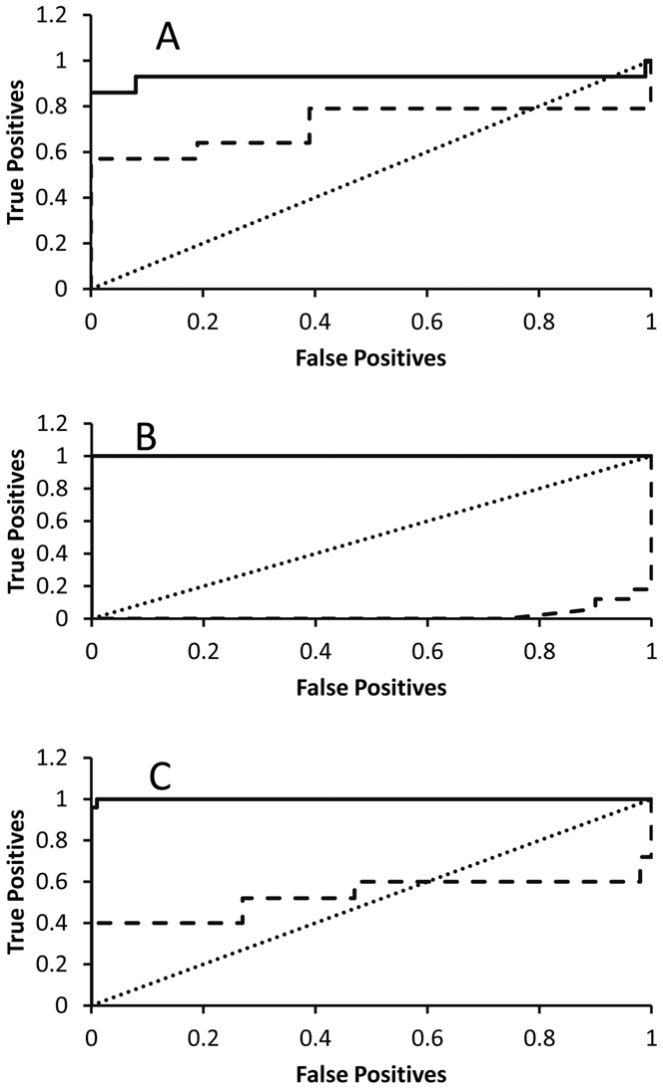
ROC curve analysis. Receiver operator characteristics (ROC) curves for analysis of internal consensus and Vina classification of native ligand and decoy complexes. A. Trypsin; B. HIV protease; C. 39 DUD proteins. Solid line, internal consensus; dashed line, Vina. A diagonal (dotted line) represents a random selection. Curves above the diagonal represent successful separation of decoys and native ligands.

Receiver operating characteristic (ROC) curves are a widely accepted way to determine accuracy of protein-ligand scoring. ROC analysis (Figure 3) indicated that the internal consensus approach performed very well on all of these sets of data. Internal consensus was more accurate than Vina in this test. The use of a discrete data set can not explain the relatively poor response of Vina on this assay, especially on the trypsin data (Figure 3b). A graph following the diagonal represents random classification on ROC curves. The performance on the ROC curve can be summarized by the area under the curve (AUC) (Table 2). For ROC AUC, a value of 0.5 or less indicates performance no better than random. A score of 1.0 indicates that candidates were ranked with all of the native complexes above the decoys. Another measure of classification accuracy is the correlation, r^2^ (coefficient of determination), between true classification and the classification of a method. The r^2^ value can be interpreted as the fraction of data variability predicted by the analysis method. An r^2^ value>0.5 indicates significant evidence of correct prediction above the random level. With both ROC AUC and r^2^ analysis, using the internal consensus strategy produced robust predictions, and was superior to Vina. The internal consensus approach also compared very favorably with surveys of other commonly used methods [2], [21].

**Table 2:** Efficiency of internal consensus analysis and Vina in classification of native ligand and decoy complexes.

Internal consensus^1^				
Protein target	ROC-AUC	s.d.	Correlation	s.d.
DUD Database	0.996	0.005	0.895	0.078
Trypsin	1.000	<0.001	1.000	<0.001
HIV-1 protease	1.000	<0.001	0.950	0.071

Vina scoring				
DUD Database	0.646	0.022	0.525	0.042
Trypsin	0.052	0.040	0.002^2^	0.001
HIV-1 protease	0.537	0.005	0.220	0.024

1 AUC and r^2^ correlation are distinct methods for scoring classification accuracy. Both have a range of 0–1 with values less than 0.5 indicating a relative lack of classification. Values were scored for independent data samples. Standard deviations, s.d., are shown.

2 r-values were negative.

As a comparison, reduced models were analyzed (Table S1). The models were: a hybrid method with three factors and neural network analysis; a hybrid method with 9 factors and linear regression analysis; an X-Score-like method with 3 factors and linear regression analysis. The three internal consensus factors most similar to those of X-score and Vina were used for models having only three factors. Internal consensus was the best model. Features specific to the internal consensus model, perhaps including both the neural network and redundant factor construction may have contributed to robust performance.

It is somewhat difficult to deconvolve the processes of a neural network that produce a score in order to understand its predictions. It is simpler, and still relevant, to determine the correlation of factors with ligand binding status. This analysis shows that different input factors contribute to the variance of complex formation for trypsin and HIV protease ligands vs. decoys (Table 3). For trypsin, VDW, hydrogen bond and coulombic factors contributed (r^2^>.65). For HIV protease, VDW, hydrogen bond and one of the hydrophobic terms (r^2^>0.64) but not the coulombic term contributed. Thus, as is evident as well from examination of the crystal structures, different types of interactions are key for these two classes of protein-ligand complexes. A possible strength of the internal consensus approach is the potential to analyze multiple representations of binding sites rather than reducing that diversity to an average as in regression methods. The high accuracy of prediction produced by internal consensus trained on multiple proteins (DUD database proteins; Figure 3, Table 2) may reflect the ability of the neural network to classify input proteins into appropriate categories. In contrast, simple correlation between the DUD database protein data set used, taken as a whole, and ligand binding shows binding correlation only with VDW terms (r^2^>0.63) and not with any of the other input factors (r^2^<0.2) suggesting that training regression models (as shown above) on multiple proteins might yield an overly simplified model. Others have suggested that empirical methods often reduce to measurement of VDW terms [26] which is consistent with our observation. The internal consensus approach performed well with this data classification task perhaps by making use of all the data available to it.

**Table 3:** Correlation between factor scores and protein-ligand complex formation.

**Factor**	**VDW1**	**VDW2**	**HB**	**HBANG**	**HBLONG**	**NHB**	**HYDR1**	**HYDR2**	**COUL**
**DUD** 1	0.640	0.697	0.108	0.183	0.015	−.028	0.071	0.080	0.178
**Trypsin**	0.671	0.553	0.713	0.732	0.681	0.782	0.450	−0.395	0.655
**HIV prt**	0.847	0.784	0.649	0.656	0.742	0.163	−0.215	0.543	0.251

1 Protein-ligand databases: DUD ligand/decoy database; Human trypsin complexes; HIV-1 protease inhibitor complexes. Correlation of factor score with ligand binding (1.0) versus decoy binding (0.0).

### Distinguishing near-native complex conformations

Scoring functions can be used to identify binding conformations, poses, of ligand that are very close to the native structures identified by x-ray crystallography. Consensus methods have been useful in identifying well-docked complexes [27]. The complexed structures identified are useful in identifying protein residues responsible for high-affinity binding or that are targets for enzyme inhibition. Root mean square deviation (RMSD) is a measure of difference in distance and conformation for two molecules. For bound ligands, a RMSD score of <2.0 Angstroms, relative to the native conformation, is generally accepted as indicating that most important contacts and protein-ligand interactions are retained [3]. The internal consensus method was trained on HIV-1 protease inhibitor data to distinguish RMSD<2.0 Angstroms complexes from decoy complexes that do not retain the natural binding site conformation.

**Figure 4:**
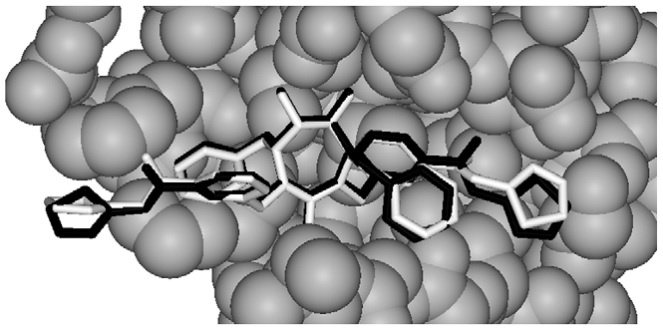
Ligand conformation selection. HIV-1 protease crystal structure 1BV7 with native ligand XV638 (gray) from the Protein Data Bank is shown with a superimposed modeled XV638 ligand (RMSD, 0.71, black) whose conformation was selected out of 45 candidate conformations by internal consensus analysis. Of the 45 conformations, 4 had RMSD values less than 2.0. Most native VDW contacts between protein and ligand are conserved (56/84 contacts with a 0.8 Angstrom threshold). Mottling of ligand occurs where the native and modeled structure are tightly aligned.

The internal consensus method classified 87.9% of 269 complexes correctly into near-native and decoy groups. The HIV-1 protease binding site is especially large and complex, holding higher molecular weight ligands [28]. An HIV-1 protease/antagonist conformation chosen by internal consensus classification with an RMSD of 0.71 Angstroms is shown in Figure 4. As is evident, modeling complexes in this manner might be useful for aspects of analysis of protein-ligand interaction. In this approach, information about known protein-ligand interactions of specific families is explicitly captured by the internal consensus neural network. Deliberately thorough training is desirable in this particular case, to force the ligand to assume a conformation as much like that of the training ligands as possible. This approach is conceptually similar to homology modeling in which the modeled protein is constrained to the structure of the target templates [29].

### Comparison of an internal consensus strategy to Vina in virtual screening

A common use for scoring methods is in the virtual screening of small compound databases to attempt to find lead molecules that might bind a target protein. This process of drug discovery requires a method to recognize target-ligand complexes that may bind tightly. Though it is common to use methods that score ligands, inevitably the use of virtual screening is to produce a short list of compounds for further testing. That is, the methods are used more to rank than to score. Here, the nonlinear function output of internal consensus analysis is used for ranking.

**Figure 5:**
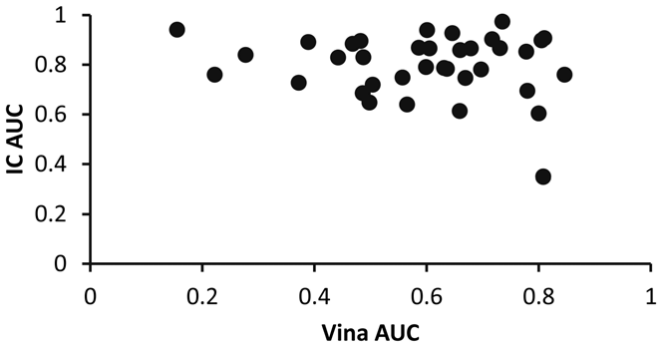
Virtual screening. Results of ROC-AUC analysis for 39 DUD protein virtual screening analyses are shown. AUC values obtained by the internal consensus method are compared to those from Vina scoring. Values above 0.5 indicate successful selection of ligands over decoys. The differences between Vina and the internal consensus method are significant (two-tailed, paired T-test; p<2.0×10^−7^).

To simulate virtual screening, documented ligands or decoys from the DUD database were docked to 39 proteins (about 70 complexes/protein). This approach has been used by a number of groups to benchmark methods for virtual screening [15], [25], [30]–[32]. The ROC AUC values resulting from internal consensus analysis were compared to those from Vina. As shown in Figure 5, internal consensus mostly produced good classifications, though a few targets did not do well. This observation suggests that the method could be used for virtual screening on most protein types.

**Figure 6:**
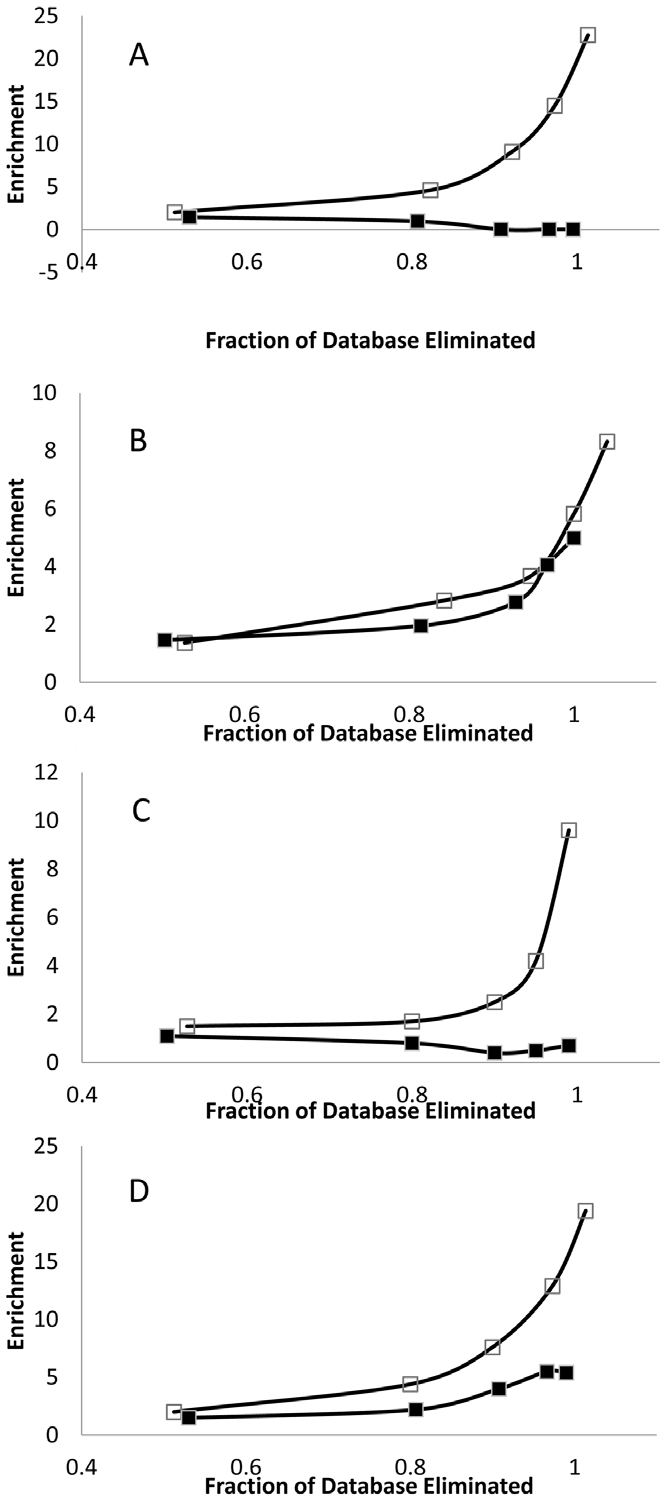
Enrichment curves. The ability of analysis by the internal consensus approach and Vina to promote ligand enrichment over decoys in virtual screening is shown. Enrichment is presented as a function of the fraction of the original database eliminated in the screen. Protein targets: A. Thymidine kinase; B. Estrogen receptor; C. Neuraminidase; D. S-adenosyl homocysteine hydrolase. An enrichment factor of 1.0 corresponds to a random selection of genuine ligands from decoys. Closed markers, Vina; open markers, internal consensus.

Virtual screening methods, crucially, must concentrate binding ligands into a small selected pool that represents only a tiny fraction of the original candidates [33]. The small, best, early, candidate pool that will be considered for further analysis must be highly enriched, because the later candidates probably will be discarded. To better analyze the ability of internal consensus analysis to promote this ‘early recognition’ of ligands, virtual screening was studied using a larger number of decoys. The method was presented with <50 ligands mixed with 1000 decoys. The pROC metric was used to determine early recognition [34], Table 4. The null distribution of pROC was used to determine statistical significance [34]. All of the tested internal consensus cases exhibited significant early recognition. Vina performed well on about half of all proteins but was less reproducible. Another measure highly relevant to virtual screening is ligand enrichment away from decoys, especially enrichment in a highly selected fraction of the ligand database. Enrichment as a function of ligand rank was performed on four protein targets, thymidine kinase, the estrogen receptor, neuraminidase and SAHH (Figure 6). When 1% of the database was selected, enrichments were 22, 7, 9 and 19-fold respectively using the internal consensus method. Maximum achievable enrichment (corresponding to recovery of only valid ligands) was 21 to 25 for this experiment.

**Table 4:** Ability of methods to reduce a large sample of mostly decoy ligands to a small sample of complexes enriched for genuine binding ligands as determined by pROC metric.

	pROC	
Protein target	Internal consensus		Vina
Trypsin	0.856*		0.513
Estrogen receptor	0.885*		0.944*
Thymidine kinase	1.493*		0.431
Retinoic acid X receptor	1.696*		2.228*
Src tyrosine kinase	0.755*		0.690
Neuraminidase	0.769*		0.451
S-adenosyl homocysteine hydrolase	1.251*		0.963*
HIV-1 protease	1.028*		0.771*

* Significant. pROC critical value (P<0.05) is 0.70 [34].

### Conclusions

Therapeutic drug development, molecular probe development and other aspects of biology make use of ligand predictions to identify important molecules. Many methods have been proposed, but none are entirely successful. Here, a new method that suggests improvements on these predictions is presented. Similar to consensus methods that combine several applications, internal consensus uses a model for ligand binding that can analyze diverse ligand binding interactions. Both the use of multiple, overlapping factors and a neural network analysis contribute to the ability of the internal consensus strategy to robustly deal with multiple types of ligand complexes. Some other models may be less complex than the data they are set to analyze. Overall, Vina showed significant ability to recognize protein-ligand complexes, but the internal consensus analysis was superior in most tests to Vina and other non-consensus methods. Internal consensus analyses can overtrain as they learn their input data, but that difficulty was formally prevented in this work. Because the internal consensus approach to scoring complexes is consistent with training on discrete binding/nonbinding data, the quantity of training data available to it may be greater than that available to some other methods, permitting more focused analyses. The internal consensus produced by this strategy may capture key aspects of ligand binding that contribute to free energy of binding. Though not formally a physics-based method, internal consensus is physics-based in spirit, attempting to provide to its neural network factors reflecting known contributions to energy. At the same time, the approach is more realistic about the messiness of real data than many methods. Docked ligands are, at best, near-native in conformation. To be useful for applications such as virtual screening, methods must be able to analyze imperfect complexes. The successes of analyses incorporating internal consensus suggests pragmatic changes in scoring methodology that might improve accuracy while making only modest compromises with existing empirical methods.

## Methods

### Factor selection

Factors were selected with an attempt to reflect physics-based interactions known to act in protein-receptor complexes. Protein-receptor or protein-decoy complexes were scored for each of the 9 factors.

Two factors were selected to reflect Van der Waals interaction. VDW1 was a Lennard-Jones related function of atomic distances:
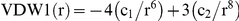
Where c_1_ and c_1_ are constants reflecting the sum of VDW distances for the atom pair to the sixth or eighth power, and r is the observed distance between the atom pair. VDW1 was truncated at 4.5 Angstroms.

VDW2 was selected to reflect more distant interactions perhaps interactions not present in the structure under consideration, but present in ensemble structures due to protein flexibility.
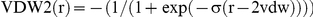
Where σ was set to 3, r is distance between an atom pair and vdw is the sum of the Van der Waals radii for the atom pair. This function is sigmoidal and reaches half maximal at r = 2vdw.

HBOND takes a value of 1 if a hydrogen bond donor is within 2.5 Angstroms of a hydrogen bond acceptor. Donors and acceptors are defined by MGLtools atom types [35].

HBANG, the hydrogen bond angle term, assumes a value of 0 if a hydrogen bond makes an angle (with hydrogen at its vertex) of less than 90°. Otherwise the value is cos(Θ)^2^, where Θ is the hydrogen bond angle.

HLONG is a term that allows long hydrogen bonds, e.g. those formed via an intervening water molecule. The conditions for permitting an HLONG interaction are the same as those for an HBOND, but the distance cutoff is HLONG<3.3 Angstroms.

NHB is a term reflecting number of hydrogen bond donors or acceptors that do not meet the criteria for forming hydrogen bonds of the HBOND or HLONG type.

HYDR1 is a term intended to correlate with a hydrophobic environment for the ligand. The value represents the number of receptor hydrophobic residues that lie within 4.5 Angstroms of the ligand [6]. Hydrophobic residues are taken as Leu, Ile, Met, Phe, Val, Tyr or Trp.

HYDR2 represents another hydrophobic term, but one based on an atomic level. The term reflects a count of the number of receptor carbon atoms within 4.5 Angstroms of a ligand carbon atom. Though only an approximation, the value of HYDRO2 may correlate with waters displaced during ligand binding.

COUL is a term for coulombic charge interactions. COUL = q_1_q_2_/r^2^ where q_1_ and q_2_ are the Gasteiger partial charges of the two atoms provided by MGLtools [35] and r is the distance between the atoms. The dielectric constant for COUL in the ligand binding pocket is not explicitly represented, but is implicit in the weighting of the term. It has been suggested that Gasteiger charges are not ideal for predicting protein-receptor interactions [36] and we have confirmed that AM1-BCC charges are superior for some proteins (not shown). However, on average Gasteiger charges seem a reasonable approximation and Gasteiger charges provided by MGLtools [35] were used for all of our analyses.

### Calculation of factor correlations

For calculation of factor correlations (r^2^), 1000 DUD decoys [24] were docked to either trypsin or HIV protease. The goal of the analyses was to determine factors with overlapping features. Correlations of factors calculated with the internal consensus methodology were considered significant if over 0.5. Docked decoy complexes were used for the correlation analyses rather than native complexes since the native series of complexes contained drugs designed to bind the enzyme active sites. Such drugs typically make highly favorable interactions of several types involving different portions of the molecule. Designed or selected compounds therefore make contacts with protein that are not independent or random, undercutting the interpretation of the correlation analyses. When native complexes were used, most factors except COUL appeared directly or indirectly correlated.

### Artificial neural network

A feedforward neural network was employed for protein-ligand complex classification using the 9 factors as inputs. A backpropagation training method was used to set network parameters. This method is essentially a steepest descent analysis to find a local minimum [13]. Using random initial parameters to perturb the training start only modestly changed the outcomes suggesting that network solutions were not highly sensitive to initial conditions. The number of training cycles for a type of analysis was determined roughly by the accuracy of the neural network predictions. For classification of native complexes training lengths were in the 200–300 cycle range. No training was extended to over 10,000 cycles even if the analysis indicated that more training might improve results. To avoid overfitting all scoring involved independent data sets. Care was taken to avoid inclusion of a ligand or decoy used in training set in the scoring set. Care was also taken to avoid excess training cycles that caused overfitting and degraded method performance (Figure 2).

### Native bound ligand examples

Ligand-receptor complexes were accessed via the Protein Data Bank or indirectly from the DUD database. Noncanonical files were corrected manually to permit use. Complexes listed in [5] were edited to remove complexes with duplicate ligands. The structural files of Table 5 were used in this study:

**Table 5:** PDBIDs of protein-ligand complexes used in analysis.

**HIV protease**
1A9M_B, 1AAQ_B, 1AJV_A, 1B6J_B, 1B6K_A, 1B6L_A, 1B6M_B, 1BDQ_B, 1BV7_A, 1C70_B, 1D4K_A, 1D4L_A, 1D4Y_A, 1DIF_B, 1DMP_B, 1G2K_B, 1G35_B, 1GNM_B, 1HBV_A, 1HIH_B, 1HOS_A, 1HPO_B, 1HPS_B, 1HPX_B, 1HSH_A, 1HVH_B, 1HVI_A, 1HVJ_A, 1HVK_A, 1HVL_B, 1HVR_A, 1HVS_A, 1HXW_B, 1KZK_A, 1MES_B, 1MSM_A, 1MTR_B, 1OHR_A, 1PRO_A, 1QBR_A, 1QBU_B, 1SBG_B, 1SDT_A, 1SH9_B, 1TCX_B, 1W5X_A, 1Z1H_A, 1Z1R_A, 1ZP8_A, 1ZPA_A, 2BPV_B, 2BPY_B, 2F80_B, 2HB3_B, 2I0A_A, 2I0D_A, 3AID_A, 7UPJ_A.
**Human trypsin**
1C1R_A, 1C5P_A, 1C5Q_A, 1C5S_A, 1C5T_A, 1CE5_A, 1F0T_A, 1F0U_A, 1G3B_A, 1G3C_A, 1GHZ_A, 1GI1_A, 1GI4_A, 1GI6_A, 1GJ6_A, 1K1I_A, 1K1L_A, 1K1N_A, 1KIM_A, 1O2H_A, 1O2J_A, 1O2N_A, 1O2O_A, 1O2S_A, 1O2W_A, 1O2Z_A, 1O30_A, 1O33_A, 1O36_A, 1O38_A, 1O3D_A, 1O3F_A, 1O3H_A, 1O3J_A, 1PPC_A, 1PPH_A, 1QB1_A, 1QB6_A, 1QB9_A, 1QBN_A, 1QBO_A, 1TNG_A, 1TNH_A, 1TNJ_A, 1TNK_A, 1TNL_A, 1V2K_A, 1V2N_A, 1V2O_A, 2BZA_A, 2FX6_A.

### Ligand docking to proteins

Ligands were docked with Vina Autodock [3]. Docking was centered on the mean coordinates of an index crystallographic ligand and extended with a 25 Angstrom radius. Torsions for ligands were calculated using MGLtools [35]. For the data here with nonredundant targets, Vina docked 50% of ligands with an accuracy of <2.0 Angstroms RMSD relative to the crystallographic conformation. This compares to published docking accuracy of Vina [3] and was adequate for this study. Vina routinely generated 9 docked poses, but only the highest-scoring pose was analyzed. On a 4 CPU PC, the average ligand docking took about 1 minute. All comparisons to Vina in this work were comparisons to the Vina scoring function only since both the Vina and the internal consensus analyses used the same Vina ligand docking conditions and the same Vina-docked protein-ligand configurations were scored (or rescored).

### ROC curves and ROC-AUC for internal consensus and Vina analyses

ROC curve analysis provided one way to judge accuracy in scoring protein-ligand complexes. For ROC curves, the true positive and false positive rates were compared as the threshold for scoring as positive was varied. ROC AUC is a simple area under the given ROC curve. The AUC value 0.5 results from a method that does not select better than a random level. Another approach to measuring prediction accuracy is the correlation coefficient, *r* ^2^ (preferred for this work over *r* which can be derived from it). Observed data for protein-ligand complexes was coded as 1 (binding, true ligand) or 0 (nonbinding, decoy). Typically, the neural network was trained using data from a single protein. Prediction values were taken as the neural network output node value (for internal consensus) or as predicted −ΔG (for Vina). In all cases the sign of the data was such that *r* would be positive if the method correctly predicted binding. Samples with approximately 60–150 native complexes and 300–750 docked decoys were created and split into training and scoring files. The training subset was used to train the internal consensus neural network. The second non-redundant sample was scored. To determine variance the process was repeated but with different training and scoring sets. In all cases the training and scoring sets did not contain any ligands or decoys in common. Training was either focused on a single target protein [18], [27] or more broadly over the DUD database [25].

### Linear regression model for scoring

To test the functioning of the internal consensus method, elements were combined in different ways. One hybrid model used the 9 input factors and a linear regression least squares training approach. A second model was designed to partially mimic the linear regression model X-score which uses three input factors. Three internal consensus input factors (VDW, HB, and HYDRO1) substituted for X-score VDW, H-bond and hydrophobicity terms. A third model used these three internal consensus factors with a neural network analysis. In each case, coefficients were estimated on one set of data. A second, non-redundant, set of data was scored using the same model that generated the coefficients.

### Virtual Screening Enrichment

Assessing virtual screening requires assessment of ligand selection. The metric pROC weights the early part of the selection curve as is desirable for screening purposes [34]. It is calculated as
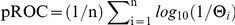
Where n is the number of true positives in the entire sample and Θ is the proportion of negatives scoring better than true positive *i*. If Θ is 0, then Θ is reset to 1/(positives+negatives) to avoid calculation problems. For pROC calculations with internal consensus analysis, the neural network output node value was used as a method score.

Enrichment is a key concept in use of scoring methods in virtual screening. Enrichment was calculated at several points at which varying amounts of the database had been discarded because it was below the score threshold. For each point the fraction of the database discarded was determined. Then enrichment was calculated:
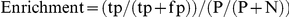
Where tp is the number of true positives in the sample remaining, fp are false positives in the sample, P is the number of positives in the original database and N is the number of negatives in the original database. Enrichment is the proportion of positives in the small selected sample divided by the original proportion of positives. The internal consensus analyses provide a non-linear output but that is not a concern, since enrichment is basically a ranking problem [37].

### Estimation of ligand binding RMSD

A group of HIV-1 protease/inhibitor complexes were analyzed by using Vina to generate a library of ligand binding poses. Complexes were analyzed using RMSD, used here as a measure of docked ligand deviation from the crystal structure ligand conformation. Docked complexes were separated into two groups: a well-docked group had an RMSD of <2.0; a decoy group had an RMSD of >3.0. The two groups (555 complexes total) were used to train the internal consensus method to distinguish complexes based on RMSD. A non-overlapping group of 269 complexes derived from HIV-1 protease bound to 32 different ligands was then scored for correct classification. To generate a series of single ligand conformations as a potential source for analysis and visualization, the ligand of PDB file 1bv7 was extracted and repeatedly docked to give 45 bound poses. Each docking generated 9 new conformations since each Vina run started its process at a configuration determined by a random seed value. Each pose was evaluated by the internal consensus method. The highest-scoring pose was documented.

### Virtual screen using DUD database of receptors and decoys

Virtual screening used the DUD database. Decoys in the database were examined especially to confirm that they were matched in features and size to ligands. DUD database decoys were similar in mean size but had somewhat less size dispersion than ligands. In general, the DUD decoys were well-matched to their ligands. Virtual screening of complexes involved screening groups of 70 or >1000 (mixes of decoys and ligands). The small groups permitted screening of more proteins. The large groups were more realistic virtual screen conditions with few true ligands and many decoys. Internal consensus analysis allows both ligand ranking and scoring. By changing the internal threshold for scoring, only higher ranked molecules are scored as positive.

## Supporting Information

Table S1 **Efficacy of hybrid methods in classifying native and decoy protein-ligand complexes.**
(DOCX)
