# Supplementary material for: Prediction of Ligand Binding Using an Approach Designed to Accommodate Diversity in Protein-Ligand Interactions
Source: PLoS One. 2011 Aug 10;6(8):e23215. doi: 10.1371/journal.pone.0023215 (PMC3157911; doi:10.1371/journal.pone.0023215)
Supplement: Table S1 — Efficacy of hybrid methods in classifying native and decoy protein-ligand complexes. (DOCX) [file pone.0023215.s001.docx]

Supporting information Table S1

**Efficacy of hybrid methods in classifying native and decoy protein-ligand complexes**

| **Protein** |  |  |  | **ROC AUC** | **S.D.** |  |  |  | **Correlation (*r*^2^)** | **S.D.** |
| --- | --- | --- | --- | --- | --- | --- | --- | --- | --- | --- |
|  |  |  |  |  |  |  |  |  |  |  |
| 3-factor, neural network model |  |  |  |  |  |  |  |  |  |  |
| DUD database |  |  |  | 0.926 | 0.027 |  |  |  | 0.599 | 0.166 |
| Trypsin |  |  |  | 0.965 | 0.032 |  |  |  | 0.851 | 0.033 |
| HIV-1 protease |  |  |  | 0.996 | 0.004 |  |  |  | 0.916 | 0.037 |
|  |  |  |  |  |  |  |  |  |  |  |
| 9-factor, linear regression model |  |  |  |  |  |  |  |  |  |  |
| DUD database |  |  |  | 0.993 | 0.006 |  |  |  | 0.739 | 0.048 |
| Trypsin |  |  |  | 0.997 | 0.005 |  |  |  | 0.771 | 0.069 |
| HIV-1 protease |  |  |  | 0.983 | 0.036 |  |  |  | 0.752 | 0.096 |
|  |  |  |  |  |  |  |  |  |  |  |
| 3-factor, linear regression model |  |  |  |  |  |  |  |  |  |  |
| DUD database |  |  |  | 0.938 | 0.024 |  |  |  | 0.230 | 0.003 |
| Trypsin |  |  |  | 0.983 | 0.007 |  |  |  | 0.482 | 0.204 |
| HIV-1 protease |  |  |  | 0.984 | 0.035 |  |  |  | 0.673 | 0.149 |
